# A Next-Generation Sequencing Data Analysis Pipeline for Detecting Unknown Pathogens from Mixed Clinical Samples and Revealing Their Genetic Diversity

**DOI:** 10.1371/journal.pone.0151495

**Published:** 2016-03-17

**Authors:** Yu-Nong Gong, Guang-Wu Chen, Shu-Li Yang, Ching-Ju Lee, Shin-Ru Shih, Kuo-Chien Tsao

**Affiliations:** 1 Department of Laboratory Medicine, Linkou Chang Gung Memorial Hospital, Taoyuan, Taiwan; 2 Department of Computer Science and Information Engineering, School of Electrical and Computer Engineering, College of Engineering, Chang Gung University, Taoyuan, Taiwan; 3 Research Center for Emerging Viral Infections, College of Medicine, Chang Gung University, Taoyuan, Taiwan; 4 Department of Medical Biotechnology and Laboratory Science, College of Medicine, Chang Gung University, Taoyuan, Taiwan; New Jersey Institute of Technology, UNITED STATES

## Abstract

Forty-two cytopathic effect (CPE)-positive isolates were collected from 2008 to 2012. All isolates could not be identified for known viral pathogens by routine diagnostic assays. They were pooled into 8 groups of 5–6 isolates to reduce the sequencing cost. Next-generation sequencing (NGS) was conducted for each group of mixed samples, and the proposed data analysis pipeline was used to identify viral pathogens in these mixed samples. Polymerase chain reaction (PCR) or enzyme-linked immunosorbent assay (ELISA) was individually conducted for each of these 42 isolates depending on the predicted viral types in each group. Two isolates remained unknown after these tests. Moreover, iteration mapping was implemented for each of these 2 isolates, and predicted human parechovirus (HPeV) in both. In summary, our NGS pipeline detected the following viruses among the 42 isolates: 29 human rhinoviruses (HRVs), 10 HPeVs, 1 human adenovirus (HAdV), 1 echovirus and 1 rotavirus. We then focused on the 10 identified Taiwanese HPeVs because of their reported clinical significance over HRVs. Their genomes were assembled and their genetic diversity was explored. One novel 6-bp deletion was found in one HPeV-1 virus. In terms of nucleotide heterogeneity, 64 genetic variants were detected from these HPeVs using the mapped NGS reads. Most importantly, a recombination event was found between our HPeV-3 and a known HPeV-4 strain in the database. Similar event was detected in the other HPeV-3 strains in the same clade of the phylogenetic tree. These findings demonstrated that the proposed NGS data analysis pipeline identified unknown viruses from the mixed clinical samples, revealed their genetic identity and variants, and characterized their genetic features in terms of viral evolution.

## Introduction

Next-generation sequencing (NGS) technology has revolutionized virus discovery [1,2] and metagenomics [3,4] in the past decade. This technology provides a considerable number of reads from different pathogens without a priori knowledge about them. These considerable number of reads are assembled into contigs by computer algorithms [5,6]. The contigs are then searched for homologs in a database (usually BLASTN [7]). The top-hit organism/species fulfilling the search criteria (eg, sequence identity and e-value threshold) is used to “label” the contig, suggesting the existence of one particular organism/species. If assembled successfully, a contig can be long enough to reveal the entire viral genome of interest. However, the de novo assembled NGS reads often yield only short contigs that are partially aligned to or even do not match the database sequences [8], possibly because the tentative virus or similar strains have never been sequenced and thus do not appear in the database or because the virus mutated considerably (through point accumulation or recombination).

The Taiwan Virology Reference Laboratory Network was established by the Taiwan Centers for Disease Control in cooperation with 13 major medical centers for surveillance of viral infections [9]. Linkou Chang Gung Memorial Hospital (CGMH) plays a key role by processing a considerable number of viral specimens from Taiwan Taoyuan International Airport and collaborating hospitals and clinics in northern Taiwan for viral cultures. For example, 52 544 specimens were collected from 2000 to 2008 for investigating epidemiologic features and virus isolation of enteroviruses alone [10]. Furthermore, CGMH is responsible for detecting emerging and re-emerging viruses (eg, H1N1 [11] and H7N9 [12]) when confronting outbreaks. However, not all viruses can be detected using routine assays such as immunofluorescence assays (IFAs).

Human rhinovirus (HRV) and parechovirus (HPeV) are 2 viruses identified in this study. HRV, a member of the family of *Picornaviridae* and assigned as species of the genus *Enterovirus* in 2008, is a positive-sense, single-stranded RNA (ssRNA) virus with an approximately 7.2-kb genome. HRV causes common cold with mild symptoms. HPeV also belongs to the family of *Picornaviridae* but is assigned to a different genus, *Parechovirus*. It is also an ssRNA virus with an approximately 7.3-kb genome. HPeV primarily causes sepsis and central nervous system (CNS) diseases in infants, and still has other unproven clinical manifestations [13]. Laboratory diagnosis of HPeV is not routinely available in clinical practice and is still evolving; antiviral therapy is also being developed [14]. In Taiwan, among hundreds of viral isolates, isolates possibly containing HPeV might not be detected using standard enterovirus identification procedures [15]. Additional investigations are therefore required to clarify the characteristics of HPeVs for more effective clinical diagnosis. Current methods for detecting HPeV include real-time polymerase chain reaction (PCR) assays of stool, blood, respiratory swabs, and cerebrospinal fluid [16]. For HPeV typing, classification of the VP1 region is the current gold standard. However, because of an unknown viral load in clinical specimens, more sensitive VP1 primers for different HPeV genotypes are required [17]. Another issue is that primers are generally not available for all HPeV genotypes [18]. NGS technology involves producing genomic sequences from clinical samples without the need for designing specific primers, thus enabling the detection of unknown or untyped HPeVs.

This study detected viruses in 42 cytopathic effect (CPE)-positive cases isolated from 2008 to 2012 and revealed their genetic diversity by using NGS. Moreover, quality reads assembled into contigs were queried against known viral sequences in the database to predict the viruses present in the clinical sample. PCR-based detection or enzyme-linked immunosorbent assay (ELISA) was used to confirm the predicted viruses. Among the catalog of possible viruses obtained from these NGS experiments, we focused on identification of HPeVs because of their reported clinical significance; furthermore, we explored their genomic context by mapping NGS reads onto them. Phylogenetic and recombination analyses were finally performed to discern the molecular evolution of the identified Taiwanese HPeVs.

## Materials and Methods

### Ethics statement

This study was approved by the Institutional Review Board (IRB) of Chang Gung Medical Foundation, Linkou Medical Center, Taoyuan, Taiwan (Approval No. 100-4378B).

### Overall methodology for NGS pipeline

**Fig 1:**
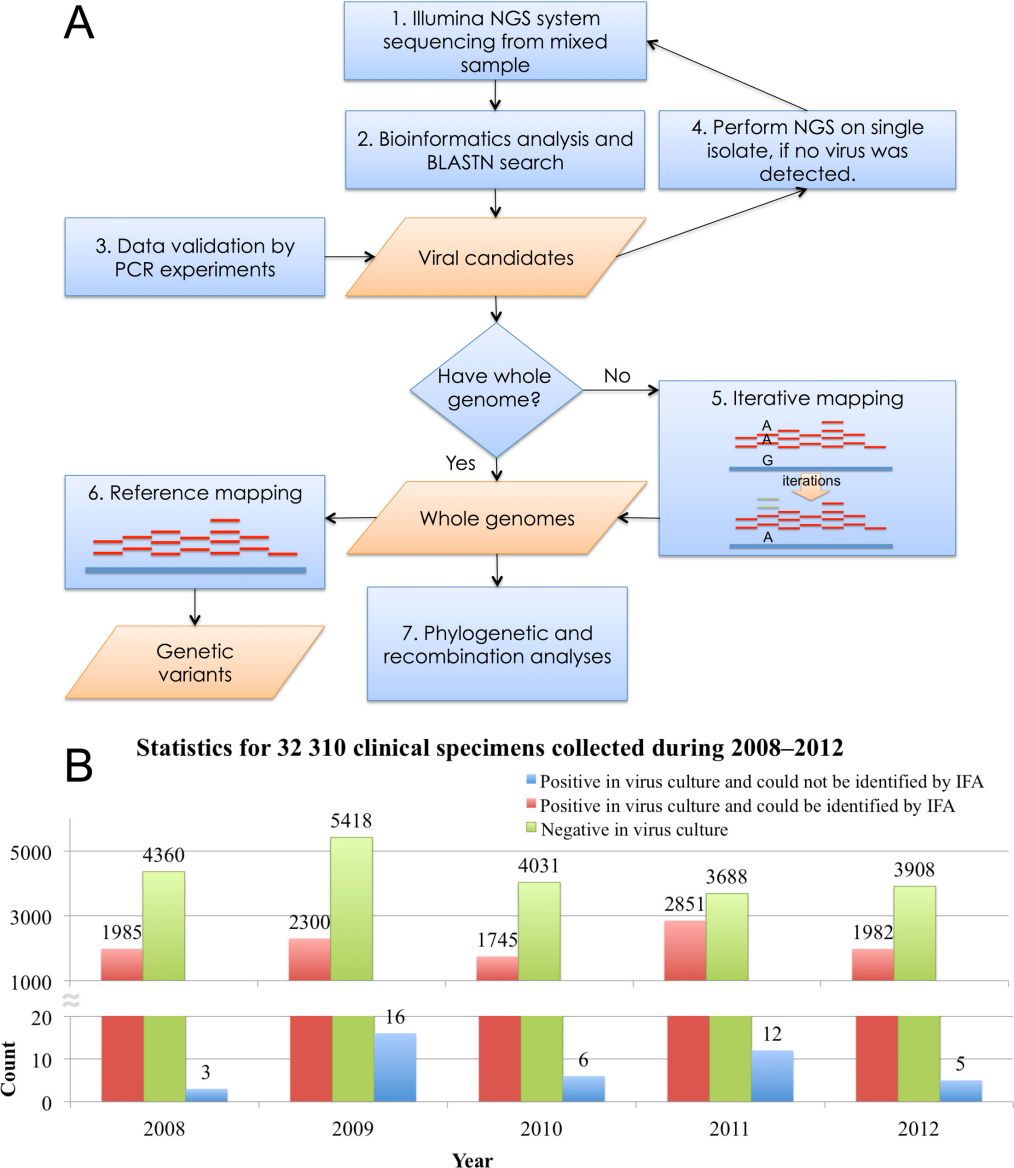
A next-generation sequencing data analysis pipeline for clinical specimens. A NGS data analysis pipeline was implemented to identify unknown pathogens, to reveal their genetic identity and variant, and to explore viral evolution from clinical samples. (A) Overall methodology. (B) Statistics for 32 310 clinical specimens collected during 2008–2012 in Linkou Chang Gung Memorial Hospital. The bar chart presents 42 cases (in blue) that could be cultured but not identified by IFA, 10 863 cases (in red) that could be cultured and identified by IFA and 21 405 cases (in green) that could not be cultured.

We implemented an NGS data analysis pipeline to identify unknown pathogens, assemble whole genomes, and investigate viral diversity from mixed/single clinical samples. Fig 1A shows the flowchart of the overall methodology. In brief, the overall methodology included the following steps: 1) NGS was performed using the Illumina system for collected clinical samples, 2) NGS reads were preprocessed using bioinformatics tools, and sequence homologs of assembled contigs were searched for BLASTN, 3) NGS diagnoses were validated using PCR or ELISA, 4) NGS analysis was performed on single isolate, if no virus was detected in PCR or ELISA confirmation, 5) an iterative mapping was implemented to obtain viral genome, if no genome was provided, 6) reference mapping was performed to identify genetic variants, and 7) genetic diversity was investigated using phylogenetic and recombination analyses. This platform is detailed in the following sections.

### Sample collection, virus isolation and RNA extraction

In total, 32 310 clinical specimens were received by CGMH for viral culturing from 2008 to 2012 (Fig 1B). Throat (TH) and nasopharyngeal (NP) swab specimens were inoculated into conventional tube cultures of MDCK, MK2, MRC-5 and RD cells. Rectal (RCT) swab specimens were inoculated into MK2, MRC-5, RD and A549 cells. These cell lines were obtained from ATCC (Manassas, Virginia, USA) and maintained in Minimum Essential Media (MEM) with 10% fetal bovine serum (FBS) and 1% antibiotic. The inoculation medium of MDCK and MK2 cells were MEM medium with 2 μg/ml trypsin and antibiotic, and of RD and MRC-5 cells were MEM medium with 2% FBS and antibiotic. Conventional culture tubes were incubated at 35°C with 5% CO2, and examined daily for CPE for two weeks. Infected cells were harvested and fixed with cold acetone for virus identification by IFA. Specific antibodies were used for detecting commonly seen viruses in clinical laboratory, as shown in S1 Table. In addition, serotype-specific monoclonal antibodies were further employed for identifying enterovirus serotypes, if specimens were enterovirus positives.

Among these 32 310 clinical specimens in this study, more than 30% of the isolates were identified by IFA as positive cases (red bar in Fig 1B) every year. However, no viruses were detected by using IFA in 42 cultured samples (blue bar in Fig 1B), including 36 TH, 2 NP and 4 RCT swab specimens. These 42 isolates were centrifuged at the maximum speed (13 500 rpm) for 10 minutes in an eppendorf microcentrifuge tube for removal of residual cells and mitochondria and filtrated through a 0.22-μm filter for improving the sensitivity and reducing the background for subsequent analyses. Viral RNA extraction was performed in 140 μL of cell culture by using the QIAamp® Viral RNA Mini Kit (Qiagen, Valencia, USA). To extract nucleic acids, carrier RNA was replaced using linear acrylamide (Life Technologies, USA) as a precipitation reagent in the QIAamp® Viral RNA Mini Kit, according to previously published methods [19]. Extracted RNA eluted using 30 μL of elution buffer was stored at −70°C. Strain names (eg, TW-00158-2011) for these 42 isolates consist of 3 parts: country of sample collection (Taiwan, abbreviated as TW), a running number (eg, 00158), and the year of isolation (eg, 2011).

### Next-generation sequencing by using the Illumina system and bioinformatics analysis

To reduce the sequencing cost, the 42 specimens were pooled into 8 groups of 5 or 6 samples. The cDNA and library of mixed samples were synthesized using Ovation^®^ RNA-Seq System V2 and Ovation^®^ Ultralow Library Systems (NuGEN, San Carlos, CA, USA) according to previously published methods [20]; these were used by the Illumina system to generate raw data of 1.5 gigabytes for each mixed sample. Preprocessing of the NGS reads was performed using CLC Genomics Workbench (version 7.5) [21] and BLASTN (version 2.2.22). In brief, we first trimmed the raw data by removing low-quality and short reads, followed by merging all the paired reads into single reads. Second, paired reads that could not be merged and merged single reads were filtered by RNA-seq analysis to remove human genome fragments. Third, the remaining reads were assembled de novo to construct contig sequences. Finally, BLASTN was used to search the assembled contigs against the nucleotide sequence (NT) database of the National Center for Biotechnology Information (NCBI) downloaded from ftp://ftp.ncbi.nih.gov/blast/db/. Viral candidates were identified using the reported top BLASTN hits for each of the queried contig sequences fulfilling the search criterion of an e-value threshold of 10^−5^. A queried contig was required to have an average read depth of at least 20, which is the recommended lower bound for revealing a whole viral genome in metagenomic NGS data [22].

### PCR or ELISA of clinical isolates

Each of the 42 clinical isolates was tested using PCR or ELISA to confirm the findings of the NGS data analysis. For PCR, viral RNA was amplified and detected using virus-specific primers or probes. Amplified products, if necessary, were sequenced using the BigDye Terminator Cycle Sequencing Kit.

Subtype-specific primers targeting the VP1 region were designed to detect HPeV-1 (primer set F1382-R3140 in Table 1). For HPeV-3 and -4, a universal primer set F1382-R3072 was designed, and the obtained VP1 sequence was queried against NT database of NCBI to determine the subtype. For HAdV detection and typing, type-specific primers and probes [23–25] were used to detect the amplified PCR product without sequencing. The detection of HRVs and echoviruses was directly performed using EV CODEHOP primers [26] to obtain the EV sequence, followed by querying the database for the serotype. For rotavirus detection, specimens were tested for the presence of the viral antigen by using the RIDASCREEN Rotavirus ELISA Kit according to manufacturer instructions.

**Table 1:** Primer designs for the HPeV genome.

Primer sets	Forward (F) and reverse (R) primers with start positions	Sequence (from 5’ to 3’)
1	F648	ACGTCTAGTGGGCCAAACC
	R2232	GAACCCCAYGAATTTTGG
2	F1382	CCCTATGTTGCWGACACAAACTA
	^a^R3140	CTGTYTTGAAAATGTCATCTGA
3	F1382	CCCTATGTTGCWGACACAAACTA
	^b^R3072	CATAGTGCTTRTARAAACCCCT
4	^a^F3022	AGAYCAGAGYCCATATG
	R4691	ACTTCCCAGCAYTCACCAGT
5	^b^F3074	GGGTTTTTAYAARCACTAT
	R4691	ACTTCCCAGCAYTCACCAGT
6	F4475	TTTATWGTWCCAATGGCACA
	R5933	GCTGGGCCCATYTTRAC
7	F4356	CWGCTAGTGAGTTYATGGAT
	R6428	TCATARATTTCCATCATGATCAT
8	F5768	ATGCAYATAGCTGGRAATGG
	R7321	TTTGGTATGTCCAATATTCCAA

This table lists the 8 primer sets used for Sanger sequencing of the HPeV genome. Their start positions were based on reference strains (accession number S45208). Superscripts a and b are marked for specific primers of HPeV-1 and HPeV-3 and -4 based on the VP1 region, respectively. Other primers without superscripts are universal primers for all three types.

^a^Specific primers for HPeV-1

^b^Specific primers for HPeV-3 and -4

### Amplifying and Sanger sequencing of the HPeV genomes

To explore the genetic variants among the identified HPeVs, we designed primers to obtain their complete coding sequences, as shown in Table 1. Viral RNA was amplified and sequenced using these primers. Eight HPeV genomes obtained in this study have been deposited in GenBank database with accession numbers KT626005 to KT626012.

### Reference mapping for genetic variant identification

To investigate the read depth and detect genetic variants of candidate viral genomes, we performed reference mapping using genomic templates with bowtie2 (version 2.2.5) [27] with default settings. Position-specific read counts and nucleotide compositions obtained after the read mapping on viral genome were examined, and a genetic variant was located where the primary nucleotide alphabet dominated less than 75% [28] and the read depth was at least 20.

### Iterative mapping for revealing viral genomes

Whole genomes by Sanger sequencing for the viruses of interest may fail due to low viral load, temperature, and so on. Therefore, no genomic template can be used for NGS read mapping. An iterative mapping procedure was implemented to construct viral genome, as follows.

Step 1 Select quality viral contigs by a depth of at least 20, and their top-hit BLASTN subjects
Step 2 Generate an initial template for reference mapping
  a If quality contigs cover whole genome, a consensus sequence of these contigs will be the initial template.
  b If quality contigs only cover partial genome, a consensus sequence of their BLASTN subjects was used for filling up missing regions. This modified sequence will be the initial template.
Step 3 Do reference mapping
Step 4 Nucleotide compositions of mapped reads at each genomic position were examined and used to substitute the nucleotide on the template sequence if a mismatch occurred. The modified template was then used as a new reference for the next round of reference mapping in Step 3 until no more substitutions were made to the template.
Step 5 Output the terminal template as target genome.

### Phylogenetic and recombination analyses

The viral sequences identified in this study, together with reference genomes retrieved from NCBI, were aligned using Clustal Omega (version 1.2) [29] for subsequent phylogenetic and recombination analyses. Phylogenetic analysis was performed using MEGA6 [30], in which an evolutionary tree was inferred using the maximum likelihood method based on the Jukes–Cantor model [31]. Initial trees for the heuristic search were obtained by applying the neighbor-joining method to a matrix of pairwise distances estimated using the maximum composite likelihood (MCL) approach. The tree was drawn to scale, with branch lengths measured in the number of substitutions per site. The bootstrap value was set to 1000. All positions containing gaps were eliminated. Recombination was detected using SimPlot (version 3.5.1) [32] with a window and step size of 600 and 20 nucleotides, respectively.

## Results

### Taxonomic classification and detection of potential viral types by using NGS

The 42 clinical specimens were pooled into 8 groups (NGS 1 to 8; Table 2). NGS was conducted for each group of mixed samples. Contigs assembled from each of these NGS experiments were cataloged into viral types by taxonomic classification based on BLASTN. In total, 361 quality viral contigs (with a depth of at least 20) from all 8 NGS experiments were queried, which predicted 47 potential viral types and their BLASTN reports as shown in Table 2 and S1 File, respectively. HRVs were detected in all these NGS data sets, except for NGS 2. In particular, NGS 4, 6, 7, and 8 only contained various subtypes of HRV type A. HPeVs of different types were observed in NGS 1, 2, 3, and 5. Other predicted viruses included echovirus type 3 (NGS 1), human adenovirus (HAdV) type 3 (NGS 1 and 2), and rotavirus (NGS 3 and 5).

**Table 2:** Preliminary NGS prediction and PCR or ELISA detection.

NGS group: Predicted viruses with genome coverage (%)	Strain names of isolates in mixed sample	PCR/ELISA detections by isolates				
		HAdV-2/3/7	HPeV-1/3/4	Rota	HRV/Echo	Final result
NGS 1: **HPeV-1 (100), Echo 3 (100), HRV-B52 (99), HRV-A21 (97), HRV-A81 (97)**, HAdV-3 (1)	TW-00158-2011	–	–		**HRV-A21**	**HRV-A21**
	TW-70307-2011	–	–		**Echo-3**	**Echo-3**
	TW-90330-2011	–	–		**HRV-B52**	**HRV-B52**
	TW-50192-2012	–	**HPeV-1**		–	**HPeV-1**
	TW-96046-2012	–	–		**HRV-A81**	**HRV-A81**
NGS 2: **HPeV-1 (95), HAdV-3 (93), HPeV-4 (82), HPeV-3 (81)**	TW-00032-2011	–	**HPeV-4**			**HPeV-4**
	TW-03067-2011	–	**HPeV-3**			**HPeV-3**
	TW-71157-2011	–	**HPeV-1**			**HPeV-1**
	TW-71170-2011	**HAdV-3**	–			**HAdV-3**
	TW-01679-2012	–	**HPeV-1**			**HPeV-1**
NGS 3: **HPeV-1 (99), HRV-A56 (98), HRV-A21 (97), HRV-B4 (64)**, HRV-A20 (44), HRV-A12 (35), Rotavirus (13)	TW-71236-2008		–	–	**HRV-A56**	**HRV-A56**
	TW-01319-2010		**HPeV-1**	–	–	**HPeV-1**
	TW-71397-2010		–	–	**HRV-B04**	**HRV-B04**
	TW-71422-2010		–	–	**HRV-A21**	**HRV-A21**
	TW-71594-2010		**HPeV-1**	–	–	**HPeV-1**
NGS 4: **HRV-A20 (97), HRV-A12 (89), HRV-A49 (89)**	TW-01662-2010				–	–
	TW-00663-2011				**HRV-A20**	**HRV-A20**
	TW-00824-2011				**HRV-A20**	**HRV-A20**
	TW-70142-2011				**HRV-A12**	**HRV-A12**
	TW-71368-2011				**HRV-A49**	**HRV-A49**
NGS 5: **HRV-A63 (97), HRV-A21 (96), HRV-A28 (87), HPeV-1 (87), Rotavirus (88)**, HPeV-4 (58), HRV-A49 (45), HPeV-3 (19), HRV-A20 (9), HRV-A12 (2)	TW-02603-2008		–	**Rota**	–	**Rota**
	TW-02680-2008		**HPeV-1**	–	–	**HPeV-1**
	TW-96061-2010		–	–	**HRV-A21**	**HRV-A21**
	TW-02547-2011		–	–	–	–
	TW-50289-2012		–	–	**HRV-A63**	**HRV-A63**
	TW-50733-2012		–	–	**HRV-A28**	**HRV-A28**
NGS 6: **HRV-A94 (98), HRV-A11 (97), HRV-A23 (96), HRV-A12 (89)**	TW-00421-2009				**HRV-A94**	**HRV-A94**
	TW-02960-2009				**HRV-A11**	**HRV-A11**
	TW-03022-2009				**HRV-A12**	**HRV-A12**
	TW-03511-2009				**HRV-A12**	**HRV-A12**
	TW-70387-2009				**HRV-A23**	**HRV-A23**
NGS 7: **HRV-A55 (97), HRV-A95 (97), HRV-A43 (94), HRV-A41 (88), HRV-A60 (86),** HRV-A11 (17)	TW-70426-2009				**HRV-A95**	**HRV-A95**
	TW-70443-2009				**HRV-A43**	**HRV-A43**
	TW-70656-2009				**HRV-A60**	**HRV-A60**
	TW-71633-2009				**HRV-A55**	**HRV-A55**
	TW-71682-2009				**HRV-A41**	**HRV-A41**
NGS 8: **HRV-A7 (97), HRV-A55 (97), HRV-A16 (94), HRV-A20 (96), HRV-A41 (91)**, HRV-A8 (58), HRV-A12 (21)	TW-71693-2009				**HRV-A16**	**HRV-A16**
	TW-71980-2009				**HRV-A20**	**HRV-A20**
	TW-72087-2009				**HRV-A41**	**HRV-A41**
	TW-72141-2009				**HRV-A55**	**HRV-A55**
	TW-72216-2009				**HRV-A7**	**HRV-A7**
	TW-72323-2009				**HRV-A20**	**HRV-A20**

A preliminary NGS prediction of potential pathogens indicates the presence of 47 viral types based on quality contigs. The coverage of 35 viral types is higher than 65% (shown in boldface font and confirmed through PCR), and that of the other 12 viral types is 58% or less. The coverage was obtained by using the covered region of contig(s) divided by the genomic length. The genomic length of HAdV was 36 000 bp, and the lengths of HRV, HPeV, and Echo were 7250 bp. The total lengths of all 11 rotavirus segments were 18 555 bp. All regions covered by one or more contigs in each segment were summed and divided by the genomic length for calculating the coverage. Depending on our NGS-predicted viruses, PCR or ELISA was performed for validation. HAdV and HPeV were confirmed using specific PCR primers and following HRV and Echo by EV CODEHOP PCR primers and rotavirus by ELISA. The final results show 40 identified viruses: 29 HRV, 8 HPeV, 1 echovirus, 1 HAdV, and 1 rotavirus.

The 361 viral contigs assembled from NGS reads ranged from 62 bp to 10 290 bp. Although few long contigs might completely or nearly span the full genome, many short ones mapped only partially to the targeted viruses. We calculated the genome coverage (%) of the mapped contigs; the coverage is shown in parentheses in Table 2. Some viruses had a coverage of 100%, such as predicted HPeV-1 and echovirus 3 in NGS 1. Only a small proportion had a low coverage. For example, HAdV-3 in NGS 1 showed only a genome coverage of 1%. In brief, 35 of the 47 identified viral types had a genome coverage of 64% or higher; these are shown in boldface font in Table 2.

### PCR or ELISA of each of 42 clinical isolates

Following the analysis of the NGS reads for the mixed samples, virus-specific molecular diagnoses for each of the 42 isolates were performed depending on the NGS-predicted viruses included in Table 2 for validating their presence. Viruses tested included HRV, HPeV, HAdV, rotavirus, and echovirus (a total of 47 viruses predicted from the 8 NGS experiments). All isolates tested positive for only one virus, except for TW-01662-2010, which tested negative for EV CODEHOP in NGS 4 where 3 HRVs were predicted, and TW-02547-2011, which tested negative for HPeV, rotavirus, and EV CODEHOP in NGS 5 where 6 HRVs, 3 HPeVs, and 1 rotavirus were predicted. The final results of molecular diagnosis for each of the 42 isolates are listed in the right-most column of Table 2. The presence of NGS-predicted viruses in Table 2 with a genome coverage of 64% and higher in the isolates was confirmed using PCR or ELISA. All predicted viruses with a genome coverage of 58% or lower tested negative by PCR or ELISA.

### NGS experiments and viral detection for 2 isolates with unknown viruses

NGS was separately implemented for the 2 isolates in Table 2 that tested negatives after all tests. For TW-01662-2010 (designated as NGS 9), 2 quality contigs (each of 2633 and 3730 bp and average depths of 35 and 49) matched positions 830 to 3462 of the HPeV-1 K129-93 genome (accession number GQ183022) and 3492 to 7077 of the HPeV-1 CAU10-NN genome (JX575746) on BLASTN. In other words, these 2 contigs encompassed most of the first half and nearly the complete second half of the HPeV-1 genome, suggesting that TW-01662-2010 is an HPeV-1 strain. Subsequently, we implemented the iterative mapping approach proposed in this study to alter the genomic content of this HPeV-1 template to obtain as many NGS reads as possible. In brief, a consensus sequence was generated from the 2 HPeV-1 genomes, which were BLASTN subjects obtained by querying the 2 contigs. This consensus sequence was used for assembling the missing regions to generate an initial template, because contigs covered nearly HPeV genome. Subsequently, the iteration process was started from this combined genome. After 5 iterations, a terminal genome was obtained with an average mapping depth of 50.1, which increased from 35 and 49 depths of two partial sequences in 2633 and 3730 bp, respectively. A total of 56 nucleotides were present in the ORF region corrected from the initial template to the terminal template sequence. Similarly, the NGS experiment (designated as NGS 10) of TW-02547-2011 resulted in one quality viral contig of 7249 bp and depth 90, which matched the HPeV-1 PicoBank strain (FM242866) in the database. An iterative procedure started with this contig (already covering the whole genome), and after 56 iterations, it ended with a contig of depth 96.7 based on the ORF region of the terminal template. No difference was observed in nucleotides between the 2 templates based on the ORF region. That is, all iterations were the cost for substituting nucleotides in 2 UTR regions. In addition, BLAST reports of 3 quality contigs from NGS 9 and 10 were summarized in S1 File.

The iterative mapping approach in this study was implemented to alter the genomic content for obtaining viral genome by attracting as many NGS reads as possible. The depths and genomic content of the terminal templates in NGS 9 and 10 seemed to be moderately corrected from their respective initial contigs though this iterative mapping process. Because the final depths in NGS 9 and 10 were 50.1 and 96.7, respectively, which were greater than the generally accepted depth threshold of 20 in exploring virus metagenomics [22], we considered that these 2 HPeV-1 viruses were present in the 2 isolates; their CDS sequences are provided in S2 File.

### Amplifying and Sanger sequencing of HPeV genomes from the isolates

Viruses detected among the 42 isolates included 27 HRV-A, 2 HRV-B, 8 HPeV-1, and 1 each of HPeV-3, HPeV-4, HAdV-3, rotavirus, and echovirus-3. Although HRVs were mostly detected in these clinical samples, we focused on the detected HPeVs and explored their genetic characteristics because of their clinical significance. The viral genome of each of the 8 PCR-confirmed HPeVs listed in Table 2 was determined using Sanger sequencing, for which 8 primer sets as shown in Table 1 were designed using the prototype strain Harris (accession number S45208). Eight genomes were obtained with lengths ranging from 6993 bp for TW-00032-2011 to 7231 bp for TW-01679-2012, covering the entire ORF sequence and the partial UTR sequence. In particular, none of these HPeV primers were applicable to the 2 HPeV-1 viruses predicted from NGS 9 and 10. These 2 HPeV-1 genomes were revealed by NGS assembly analysis. In summary, the genetic characteristics of 10 HPeV genomes, including 8 determined by Sanger sequencing and 2 assembled from NGS analysis, were investigated.

### Read depth and genetic variants of HPeV genomes

**Fig 2:**
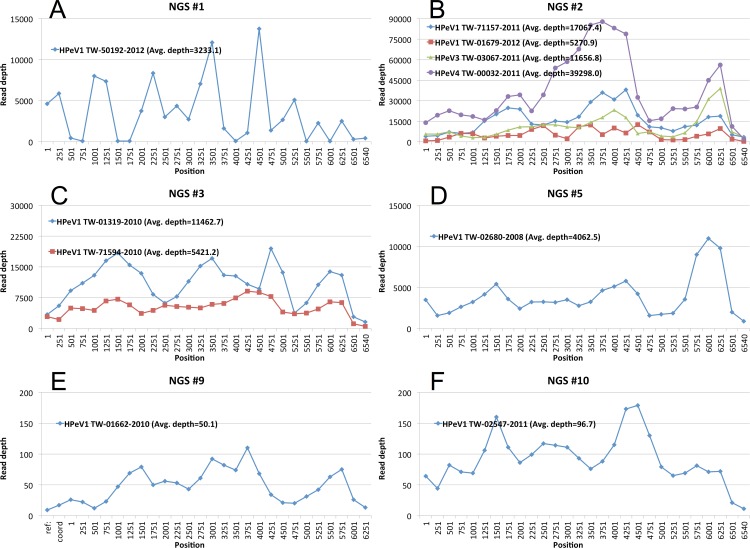
Read distributions of HPeV genomes revealed in NGS experiments. (A)–(D) Read distributions for 8 genomes obtained using PCR. NGS data sets 1, 2, 3, and 5 were each mapped to 1, 4, 2, and 1 HPeV genomes, respectively, generated using PCR. (E) and (F) Read distributions for 2 genomes revealed by NGS contig assembly, BLASTN search, and iterative mapping (as described in the Materials and Methods section).

NGS reads from each of the 10 experiments were respectively mapped to each of the 10 HPeV genomes, including 8 obtained by PCR and the 2 from analyzing NGS reads alone. This was performed to assess the overall depth distribution along the genome and to identity genetic variants exhibiting heterogeneous nucleotide compositions. Fig 2A–2F shows the read coverage distribution along the ORF of the reported HPeV genomes for NGS 1 (one HPeV-1 strain), 2 (two HPeV-1 strains, and one strain each of HPeV-3 and HPeV-4), 3 (two HPeV-1 strains), 5 (one HPeV-1 strain), 9 (one HPeV-1 strain), and 10 (one HPeV-1 strain), respectively. The depth distributions in Fig 2A–2D showed non-zero coverage across the entire ORF, and an average depth of at least 3000 was obtained. The 2 genomes obtained from assembling NGS reads and iterative mapping also showed non-zero distributed depth coverage in the ORF (Fig 2E and 2F), although their depths were much more shallow than those were observed in the other 8 HPeV genomes.

**Fig 3:**
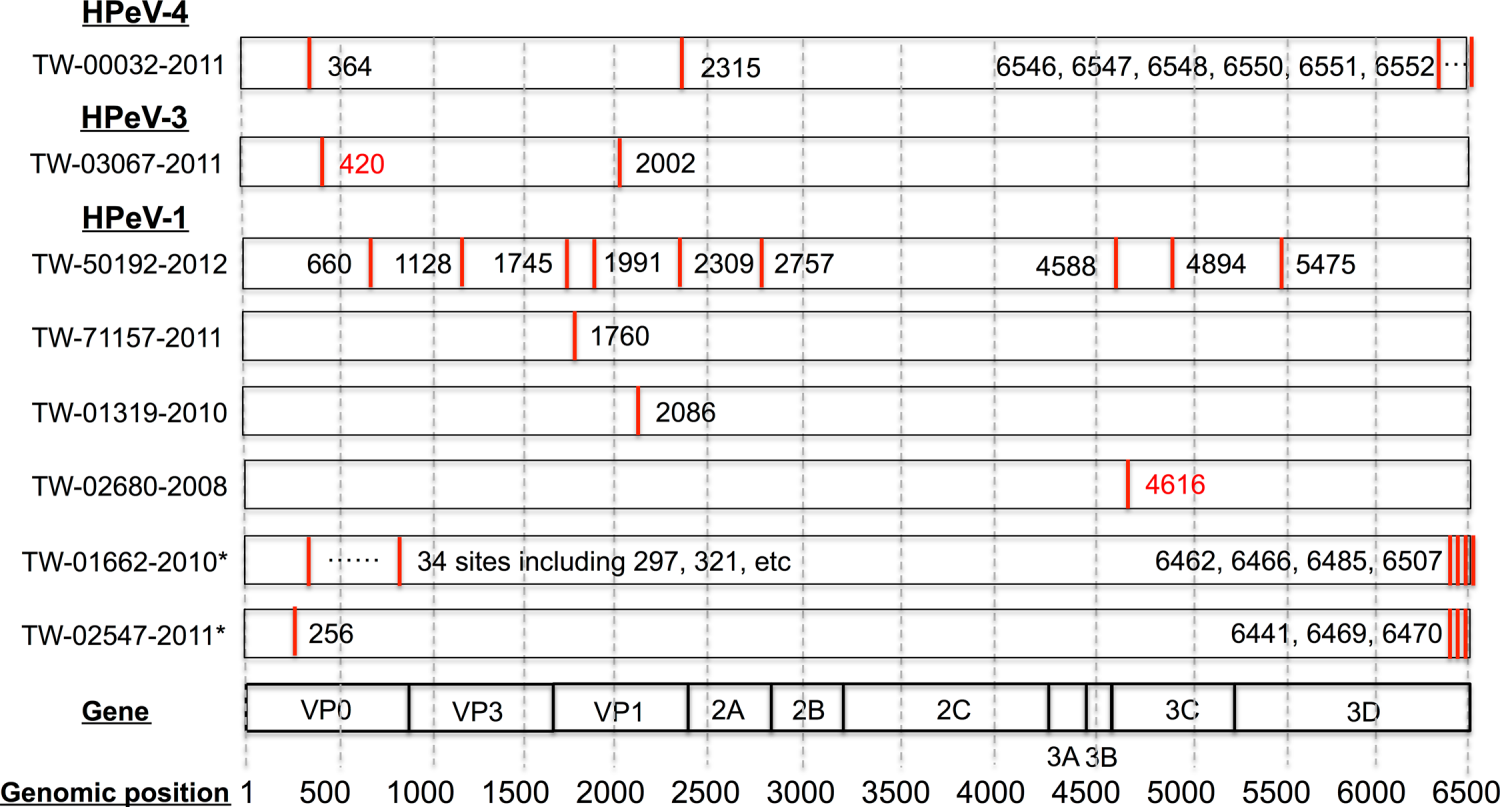
Genetic variants with heterogeneous genetic populations. In total, 64 genetic variants were detected from 6 HPeV-1 (asterisks indicate 2 predicted genomes), 1 HPeV-3, and 1 HPeV-4 genomes, including 38 in VP0, 14 in 3D, 7 in VP1, 3 in 3C, 1 in 2A, and 1 in VP3. Among 64 variants, 8 and 2 were detected from HPeV-4 and HPeV-3, respectively, and the others from HPeV-1. Two nonsynonymous mutations at positions 4616 in HPeV-1 (TW-02680-2008) and 420 in HPeV-3 (TW-03067-2011) are marked with red text.

We examined the mapped reads on each of the 10 genomes in Fig 2 and revealed their genetic variants. As mentioned earlier, a genetic variant was defined as a nucleotide position where the primary nucleotide alphabet appeared in less than 75% of the mapped reads with a read depth of at least 20. Fig 3 shows the genomic locations based on CDS that were graphed by the HPeV type. The HPeV-4 genome (TW-00032-2011) contained 8 variants: 1 in VP0, 1 in 2A, and 6 others at the 3’ end of 3D. The HPeV-3 genome (TW-03067-2011) displayed 2 variants: 1 in VP0 and 1 in VP1. The one at position 420 of VP0 (marked in red in Fig 3) consisted of A (74.6%) and C (25.1%) and is a nonsynonymous substitution leading to an amino acid change from Glu to Asp. Six of the eight TW HPeV-1 genomes collectively displayed 54 genetic variants, among which 42 were abundant in VP0 and 3D in 2 in-silico assembled genomes (TW-01662-2010 and TW-02547-2011 in Fig 3) from NGS. The other 12 genetic variants were distributed on 4 TW HPeV-1 genomes obtained Sanger sequencing. The other nonsynonymous substitution at the nucleotide position 4616 in 3C of TW-02680-2008 consisted of C (69.1%) and T (30.4%) and led to an amino acid change from Pro to Leu. S3 File provides comprehensive information of these genetic variants, including their Sanger sequencing bases, NGS read depths and compositions, NGS read consensus in nucleotides, and the translated amino acids from the corresponding codons containing these genetic variants.

### Genetic evolution of HPeV genomes

All HPeV genomes from NCBI were retrieved in March 2015 for assessing the sequence diversity and evolution of the 10 TW HPeV strains obtained in this study. The multiple sequence alignment revealed one distinct 6-bp deletion at positions 97 to 102 (in VP0 based on HPeV-1 CDS of 6540-nt long) in the HPeV-1 strain TW-02680-2008. This deletion resulted in a missing 2-aa short peptide “GN” in the translated protein sequences, as shown in S1 Fig.

**Fig 4:**
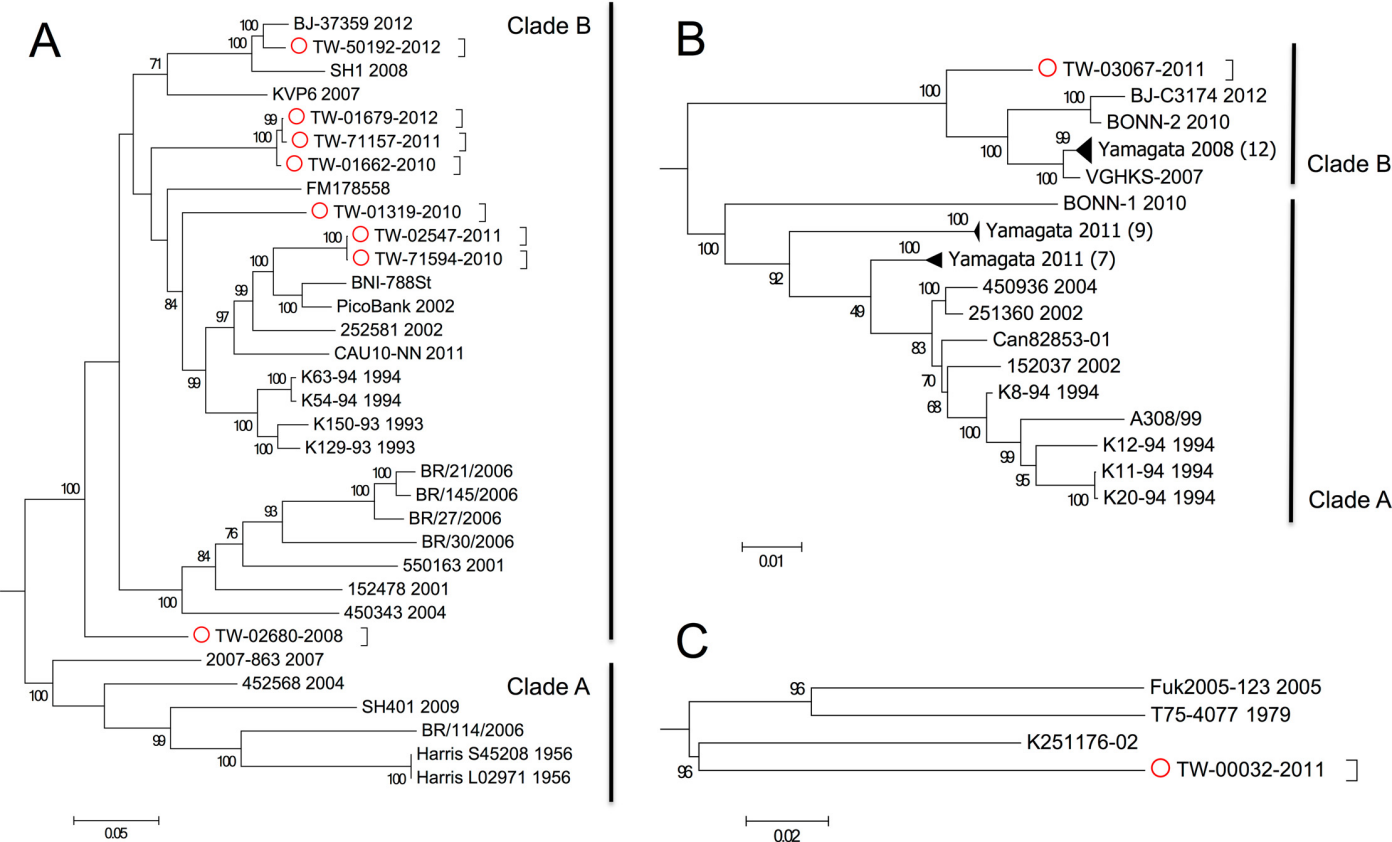
Phylogenetic analysis of genomes of HPeV types 1, 3, and 4. Genomes of all HPeV types 1, 3, and 4 from NCBI were retrieved in March 2015 for phylogenetic analysis with the 10 genomes (red circles) obtained in this study. (A) 33 HPeV-1 ORF sequences, including 8 from this study, were used to generate a tree branching into Clades A and B. (B) 42 HPeV-3 ORF sequences, including one from this study (TW-03067-2011), were used to generate a tree branching into Clades A and B. The 3 groups of Yamagata strains were compressed by triangles; their sequence counts are shown in parentheses. (C) 4 HPeV-4 ORF sequences, including one from this study (TW-00032-2011), were used to generate a tree.

Fig 4A shows the phylogenetic tree based on 33 complete HPeV-1 ORF sequences, including the 8 TW sequences identified in this study. The most distinct pairwise percent identity among all 33 HPeV-1 sequences (or 32 sequences excluding the Harris strain isolated in 1956) was 79%, demonstrating genetic heterogeneity within this particular virus within a span from 1956 (or 1993) to 2012. All TW HPeV-1 genomes were classified into Clade B, within which the pairwise percent identities were 86% or higher, suggesting that TW strains were similar to the already published Clade-B viruses in the database. One of the TW strains, TW-02680-2008, showed an average identity of 86.3%. Similarly, comparing the other TW strains to the Clade-B strains, 7 average identities were from 87.3% to 88% and were slightly stronger than that of TW-02680-2008 (86.3%). TW-02680-2008 was distantly located from the other 26 Clade-B viruses in Fig 4A. A phylogenetic tree of TW-03067-2011 with other 41 HPeV-3 viruses retrieved from database is graphed in Fig 4B. The most distinct pairwise percent identity among all these HPeV-3 sequences was 88%. This demonstrated that HPeV-3 viruses exhibited less selection pressure in a similar span from 1994 to 2012 in comparison with HPeV-1 population, which displayed a much smaller percent identity of 79%. TW-03067-2011 had least 95% identity to the other HPeV-3 viruses in Clade B in Fig 4B. Fig 4C shows the tree for an HPeV-4 strain (TW-00032-2011) identified in this study with 3 HPeV-4 reference genomes available from NCBI. TW-00032-2011 showed 83.0%–83.6% identity compared with that of the 3 references.

### Genetic recombination of HPeV genomes

**Fig 5:**
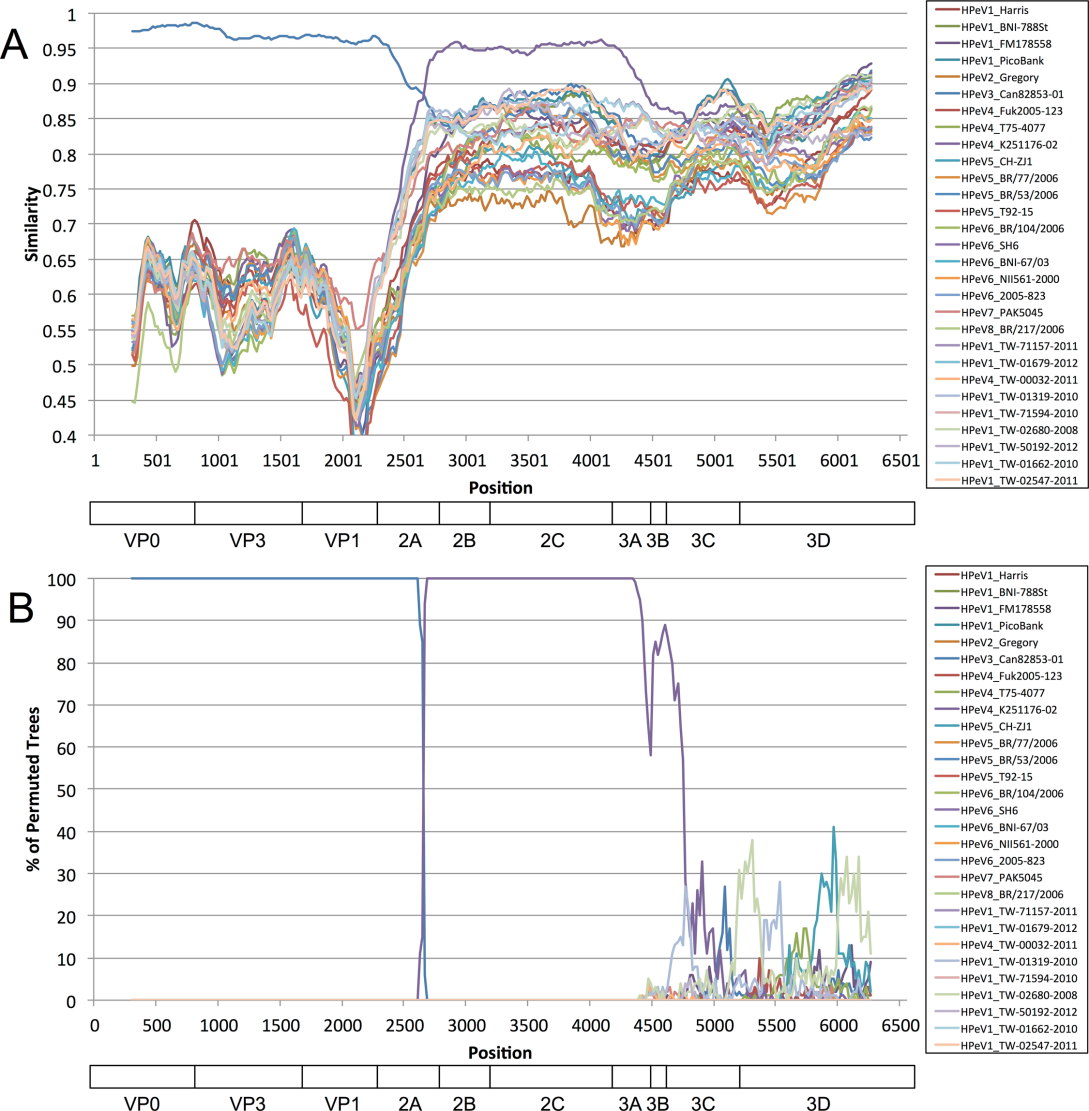
Recombination analysis of HPeV genomes. Twenty HPeV references from NCBI were retrieved in March 2015 for recombination analysis with the 10 genomes obtained in this study. (A) SimPlot and (B) Bootscan analyses of 30 HPeV ORF sequences. The query sequence was set to TW-03067-2011 of HPeV-3, which was found to resemble an HPeV-4 strain (K251176-02) from the C-terminal 2A to N-terminal 3C region.

Frequent recombination has been reported for the family of *Picornaviridae*. We performed Simplot and Bootscan analyses on the 10 TW HPeV viruses with 20 reference genomes encompassing all 8 known HPeV subtypes. The similarity plot of TW-03067-2011 (HPeV-3) resembled a HPeV-3 reference sequence (Can82853-01) in VP0, VP3, and VP1, with identity of approximately 95%–99% (Fig 5A). Thereafter, the percent identities reduced significantly to less than 88% for Can82853-01. However, an HPeV-4 strain (K251176-02) was found to have more than 96% percent identity than that of this TW HPeV-3 in the middle of the ORF spanning the C-terminal 2A to N-terminal 3C region. A Bootscan plot in Fig 5B clearly illustrates the switch over of the 2 HPeV types. Notably, the other members of Clade B also showed recombination with this HPeV-4 strain in this region, including BJ-C3174, BONN-2, VGHKS-2007, and 12 Yamagata/2008 strains (data not shown).

## Discussion

NGS technology can produce genomic sequences without the need for designing specific primers. Hence, this technology is useful for viral detection in clinical samples. A considerable number of epidemiology samples from northern Taiwan are received by CGMH for viral culturing every year; 52 544 clinical specimens were received from 2000 to 2008 [10] and 32 310 were received in the study period (2008 to 2012). CPE-positive isolates could not be identified using IFA, which might contain novel or emerging viruses that are public health risks, such as MERS-CoV, Ebola, SARS, and avian flu [33]. In addition, unknown pathogens might also exist in our culture-negative cases (approximately 60% of 32 310 clinical samples). A study reported that viral pathogens from 1406 of 2259 (approximately 60%) collected samples of patients with serious illness could also not be detected [34]; new diagnostic tests were required for those culture-negative cases.

Table 2 presents the 47 viral candidates detected using NGS, of which 35 candidates were confirmed using PCR. Among these 35 candidates, most had genome coverage higher than 80%, and only one (HRV-B4 in NGS 3) had a genome coverage of 65%. The other 12 candidates were not confirmed using PCR; their genome coverage was lower than 59%. As expected, higher genome coverage indicates that these viral genomes were well represented by the assembled contigs, and lower coverage makes confirming the existence of these viruses challenging. Although the HRV-B4 candidate in NGS 3 showed a lower coverage of 64% than that of the other 34 candidates, one contig having an extremely high depth of 10 043 with 4743 bp was categorized into this viral type, suggesting the existence of this HRV in NGS 3. By contrast, the HRV-A8 candidate in NGS 8 showed a coverage of 58%. Only one contig having an average depth of 29 with 4212 bp was categorized into this viral type. The top 3 BLAST subjects of this contig were HRV-A8, -A95, and -A45 sequences. Sequence similarities between viral pathogens in mixed sample may result in a false positive rate when only considering the top one hit as the potential viral candidate.

Because 3–16 CPE-positive cases were received every year, we pooled samples for NGS analysis to reduce the sequencing cost. However, NGS analysis of mixed samples has the limitation to assign viral candidates to each isolate after BLASTN searches of quality contigs. Hence, NGS-identified viruses were confirmed and assigned to each isolate by using PCR/ELISA. All but 2 isolates (TW-01662-2010 and TW-02547-2011) were validated. An iterative mapping procedure was further performed for these two isolates that were not identified using PCR/ELISA to reveal viral genomes for detecting unknown pathogens. Although the read depths of these 2 genomes were lower than those of viral genomes from the other NGS data set, this iterative mapping process could be useful to detect unknown pathogens in clinical samples with a low viral load (or low CPE) in clinical samples. In addition, viral culturing with a low viral load negatively affects NGS experiments. Based on our experience, the time required for HPeV culture by using the RD or MRC-5 cell line is usually 3 weeks, which is much longer than 1 week for enterovirus, a common virus in regular clinical/virology laboratories. Although HPeVs have been replicated on the RD99, A549, and vero cell lines, HPeV culture is still limited by the low induction of CPE [35].

To compare current NGS pipelines including VirusFinder [36] and VirFind [37], the novelty of the proposed pipeline is to identify unknown pathogens in a mixed clinical sample, and to implement an iterative mapping for generating viral genomes. In terms of viral detection, VirusFinder could detect unknown pathogens in a single sample, but not a mixed one. The assignment of viral candidates for each sample in the proposed pipeline was by using PCR or ELISA experiments, and in VirFind by using a degenerate oligonucleotide primed RT-PCR method with multiple barcodes. The design of barcoded primers was only suitable for detecting known viruses in target samples. Moreover, an iterative mapping was implemented in this study for constructing whole genome to improve short contigs. These two pipelines did not deal with the issue of short contigs.

In this study, we used HPeV as an example to investigate the genetic diversity of viral genomes. This virus is highly infectious and causes a variety of symptoms in early childhood [38] and has atypical molecular and biological properties [13]. This study is the first to report the three genetic diversities of Taiwanese HPeV strains through various analyses, as follows. First, the phylogenetic tree in Fig 4A indicated that one HPeV-1 virus (TW-02680-2008) was highly divergent from the rest of Clade B, in which 6 nucleotide deletions were found in contrast to the other HPeV-1 viruses from the database. Although the biological consequences of this deletion are unknown, this deletion in VP0 has not been reported. Second, we suggest two cut-off values for the identification of genetic variants in a mixed sample with 1.5 gigabytes of raw reads, including a primary nucleotide composition of less than 75% and a minimum depth of 20. Based on these cut-offs, 64 genetic variants were detected from 10 HPeV strains by using NGS read mapping for revealing viral quasispecies/heterogeneity. Although additional investigations for exploring the biological consequences of these genetic variants from HPeV are required, genetic variants in the form of a viral quasispecies located in antigenic domains or drug resistant markers were similarly identified from influenza viruses. One study revealed nucleotide heterogeneity as quasispecies for characterizing influenza A/H1N1/2009 virus by using NGS read mapping [39]. Two specific amino acids (Gly172Glu and Gly239Asn) with 25% heterogeneous populations were identified, which were located on the Sa and Ca2 antigenic sites on the hemagglutinin (HA) gene, respectively. Another study reported that Arg292Lys, one of markers for N2 that reduces oseltamivir susceptibility, was a viral quasispecies [40]. Finally, recombination events among HPeV types 1, 4, 5, and 6 were frequently detected within nonstructural (NS) genes, similar to many picornaviruses, but were more restricted among HPeV-3 strains [41–43]. Our HPeV-3 strain (TW-03067-2011) was found to be recombinant with a known HPeV-4 (K251176-02). Fig 5 illustrated the switch over of the 2 HPeV types in 2A. The similarity plot of TW-03067-2011 resembled an HPeV-3 reference (Can82853-01) from VP0 to VP1, and K251176-02 from 2B to 3C. Although it was reported that this HPeV-4 strain may have a recombination event with 2 HPeV-3 strains (A308/99 and Can82853-01, as shown in Clade A of Fig 4B) in the P2–P3 region [44], our findings further indicated that HPeV-3 strains in Clade B were recombinant with this HPeV-4, showing a genomic region from the C-terminal 2A to N-terminal 3C derived from HPeV-4. In conclusion, we established the baseline information of NGS technology for viral detection and explored genetic diversities and signatures of HPeVs by the proposed NGS data analysis pipeline. The results of this study may help to improve epidemiological surveillance and investigations in Taiwan and around the world.

## Supporting Information

S1 Fig Deletions in VP0 gene in TW-02680-2008. Deletions in VP0 in TW-02680-2008 are marked using a red rectangle, located at positions 33 and 34 in amino acid (or from 97 to 102 in nucleotides) based on the VP0 gene. The other HPeV-1 strains in this study and the database show “GN” at these amino acid positions.
(PDF)

S1 File BLAST reports of quality viral contigs with a depth of at least 20. 364 BLASTN reports of quality viral contigs from 10 NGS experiments were summarized. The numbers of quality contigs from NGS 1 to 10 are from 7, 28, 19, 35, 48, 62, 50, 112, 2 and 1, respectively.
(XLSX)

S2 File Two coding sequences of TW-01662-201 and TW-02547-2011. Two isolates (TW-01662-201 and TW-02547-2011) tested negative for PCR. An iterative mapping method was implemented to generate their coding sequences.
(FASTA)

S3 File Sanger sequencing bases, NGS read consensuses, and depths and compositions of 64 genetic variants. A genetic variant was defined as a nucleotide position having its primary nucleotide alphabet appearing in less than 75% of the mapped reads with read depth of at least 20. A total of 64 variants were identified using these 2 criteria. In total, 38 were found from TW-01662-2010, 9 from TW-50192-2012, 8 from TW-00032-2011 (HPeV-4), 4 from TW-02547-2011, 2 from TW-03067-2011 (HPeV-3), 1 each from TW-71157-2011, TW-01319-2010, and TW-02680-2008, and none from TW-01679-2012 and TW-71594-20110. For example, in TW-03067-201, Sanger sequencing base and NGS read consensus at position 420 in VP0 gene are “C” and “A,” respectively. This nonsynonymous substitution causes an amino acid change from Glu to Asp. Read depth at this position is 6219, including 4638 As, 1562 Cs, 15 Gs, 3 Ts, and 1 gap. In addition, 2 predicted genomes identified using iterative mapping are marked with asterisks.
(XLSX)

S1 Table A summary of commercial antibodies for viral screening and identification. 5 kits for viral screening and identification were used, which contain specific antibodies for commonly seen viruses in clinical laboratory.
(DOCX)
