# Supplementary material for: A Next-Generation Sequencing Data Analysis Pipeline for Detecting Unknown Pathogens from Mixed Clinical Samples and Revealing Their Genetic Diversity
Source: PLoS One. 2016 Mar 17;11(3):e0151495. doi: 10.1371/journal.pone.0151495 (PMC4795770; doi:10.1371/journal.pone.0151495)
Supplement: S1 Fig — Deletions in VP0 in TW-02680-2008 are marked using a red rectangle, located at positions 33 and 34 in amino acid (or from 97 to 102 in nucleotides) based on the VP0 gene. The other HPeV-1 strains in this study and the database show “GN” at these amino acid positions. (PDF) [file pone.0151495.s001.pdf]

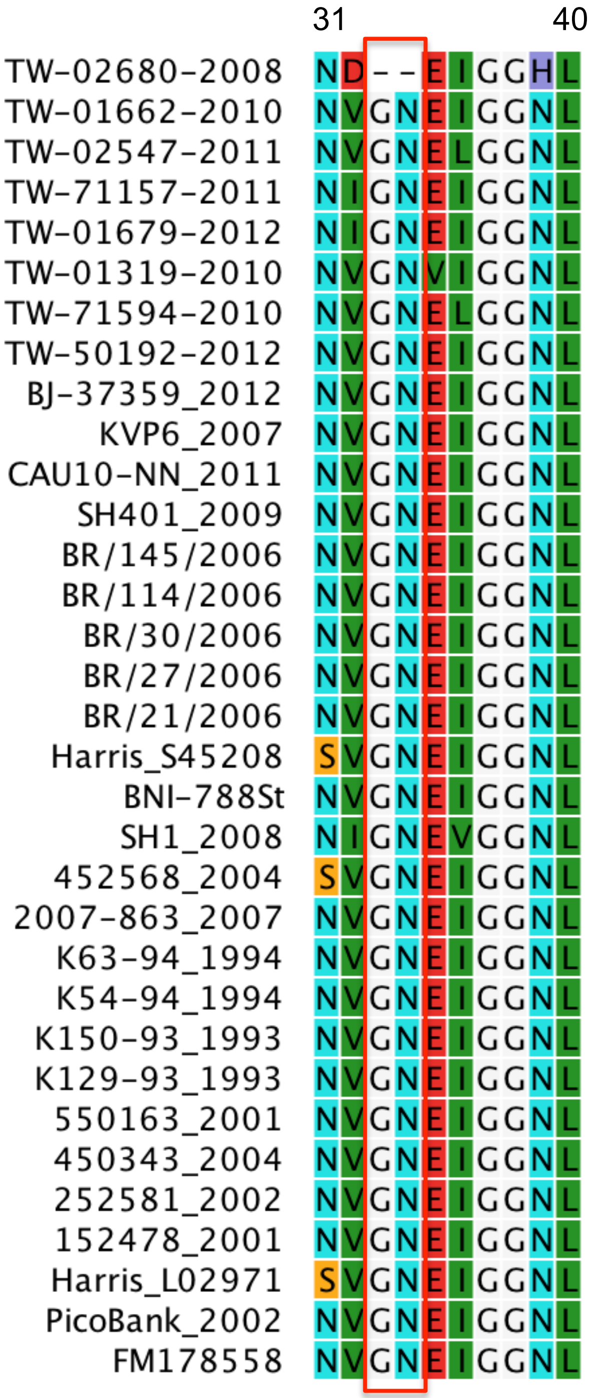
31
40
TW-02680-2008 ND--EIGGHL
TW-01662-2010 NVGNEIGGNL
TW-02547-2011 NVGNELGGNL
TW-71157-2011 NIGNEIGGNL
TW-01679-2012 NIGNEIGGNL
TW-01319-2010 NVGNVIGGNL
TW-71594-2010 NVGNELGGNL
TW-50192-2012 NVGNEIGGNL
BJ-37359_2012 NVGNEIGGNL
KVP6_2007 NVGNEIGGNL
CAU10-NN_2011 NVGNEIGGNL
SH401_2009 NVGNEIGGNL
BR/145/2006 NVGNEIGGNL
BR/114/2006 NVGNEIGGNL
BR/30/2006 NVGNEIGGNL
BR/27/2006 NVGNEIGGNL
BR/21/2006 NVGNEIGGNL
Harris_S45208 SVGNEIGGNL
BNI-788St NVGNEIGGNL
SH1_2008 NIGNEVGGNL
452568_2004 SVGNEIGGNL
2007-863_2007 NVGNEIGGNL
K63-94_1994 NVGNEIGGNL
K54-94_1994 NVGNEIGGNL
K150-93_1993 NVGNEIGGNL
K129-93_1993 NVGNEIGGNL
550163_2001 NVGNEIGGNL
450343_2004 NVGNEIGGNL
252581_2002 NVGNEIGGNL
152478_2001 NVGNEIGGNL
Harris_L02971 SVGNEIGGNL
PicoBank_2002 NVGNEIGGNL
FM178558 NVGNEIGGNL
