# Supplementary material for: A Next-Generation Sequencing Data Analysis Pipeline for Detecting Unknown Pathogens from Mixed Clinical Samples and Revealing Their Genetic Diversity
Source: PLoS One. 2016 Mar 17;11(3):e0151495. doi: 10.1371/journal.pone.0151495 (PMC4795770; doi:10.1371/journal.pone.0151495)
Supplement: S1 Table — 5 kits for viral screening and identification were used, which contain specific antibodies for commonly seen viruses in clinical laboratory. (DOCX) [file pone.0151495.s005.docx]

**S1 Table. A summary of commercial antibodies for viral screening and identification.**

| Commercial antibody kit (Company, Country) | Viruses |
| --- | --- |
| D^3^ Ultra DFA Respiratory Virus Screening and ID Kit (Diagnostic Hybrids, USA) | Influenza A/B viruses, respiratory syncytial virus (RSV), human adenovirus (HAdV), human parainfluenza viruses (hPIVs) in types 1−3, and human metapneumovirus (hMPV) |
| LIGHT DIAGNOSTICS™ Pan-Enterovirus Reagent (Merck Millipore Corp., Germany) | Enterovirus (EV) |
| LIGHT DIAGNOSTICS™ VZV Antibody FITC Reagent (Merck Millipore Corp., Germany) | Varicella-zoster virus (VZV) |
| Anti-Herpes Simplex 1/2 Argene (bioMérieux Clinical Diagnostics, France) | Herpes simplex virus (HSV) |
| Anti-cytomegalovirus IEA Argene (bioMérieux Clinical Diagnostics, France) | Cytomegalovirus (CMV) |
